# Performance metrics outperform physiological indicators in robotic teleoperation workload assessment

**DOI:** 10.1038/s41598-024-82112-4

**Published:** 2024-12-28

**Authors:** Gift Odoh, Aleksandra Landowska, Emily M. Crowe, Khairidine Benali, Sue Cobb, Max L. Wilson, Horia A. Maior, Ayse Kucukyilmaz

**Affiliations:** 1 School of Computer Science, University of Nottingham, Nottingham, UK; 2 School of Psychology, University of Nottingham, Nottingham, UK; 3 Faculty of Engineering, University of Nottingham, Nottingham, UK

**Keywords:** Computer science, Information technology

## Abstract

Robotics holds the potential to streamline the execution of repetitive and dangerous tasks, which are difficult or impossible for a human operator. However, in complex scenarios, such as nuclear waste management or disaster response, full automation often proves unfeasible due to the diverse and intricate nature of tasks, coupled with the unpredictable hazards, and is typically prevented by stringent regulatory frameworks. Consequently, the predominant approach to managing activities in such settings remains human teleoperation. Teleoperation can be demanding, especially in high-stress situations, and involves a complex blend of both cognitive and physical workload. We present an experiment to explore a range of physiological and performance-related metrics for workload assessment during robotic teleoperation. Thirty-five participants performed a teleoperation task, during which we manipulated cognitive and physical workload conditions. We recorded multiple metrics, including brain activity using functional Near-Infrared Spectroscopy, galvanic skin responses, cardiovascular responses, subjective workload ratings, task and robot performance data. Our results suggest that robotic teleoperation performance may be the most robust metric for distinguishing between different levels of workload experienced during teleoperation, with most physiological measures becoming insignificant to distinguish high cognitive workload.

## Introduction

**Fig. 1:**
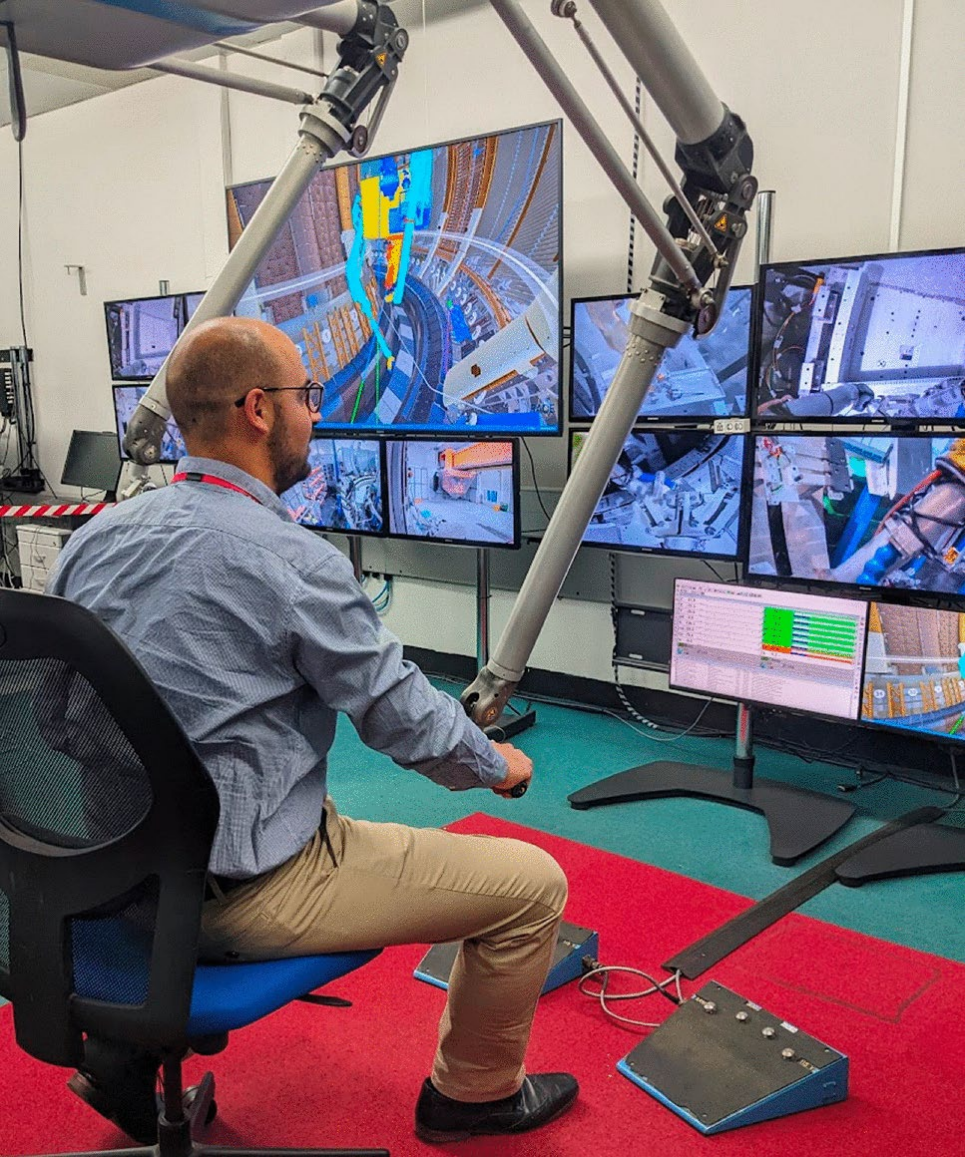
A representative leader-follower teleoperation system used by the nuclear industry involves a human operator using a bimanual interface to control two identical arms situated in a remote location. The operator must monitor the scene through multiple displays and communicate with others in the control room to coordinate the operation.

Teleoperation is widely used to control robots in environments that are dangerous or difficult for humans to access safely, such as nuclear waste decommissioning^1^, space robotics^2^, underwater robotics^3^, and surgery^4^. These systems frequently utilise leader-follower manipulators with joint-to-joint mapping to offer intuitive and convenient control interfaces for human operators^5^ (Fig. 1). However, teleoperation presents several challenges for human operators. These challenges include suboptimal display systems and limited views from static cameras, leading to *blind operation*; difficulty in controlling heavy objects against gravity; unergonomic postures due to extensive motion ranges of local (leader) manipulators; and need to coordinate with other humans in the control room. These issues increase both the physical and cognitive effort required from operators, causing fatigue and poor operation^6,7^. Operators are also challenged to adapt to a fundamentally different sensorimotor control loop, relying on limited visual feedback from cameras^8^.

The resulting high cognitive demand in teleoperation places significant strain on operators^9^, underscoring the need for system-based assistance to operators experiencing high levels of mental workload^10–12^. While technological progress has facilitated the development of fully autonomous robotic systems in controlled settings, many teleoperation applications resist automation due to the essential requirement for human problem-solving abilities and dexterous manipulation skills^8^. Additionally, stringent regulatory requirements in safety-critical domains hinder the adoption of autonomous robotic approaches^13^. As a result, human-in-the-loop assistance mechanisms are preferred, in the form of shared control—merging operator and system inputs^14–16^—or traded control—alternating control between operator and system^17–20^. Both paradigms require trigger mechanisms to determine when robot autonomy should intervene. Although real-time workload monitoring can be used for the application of these assistance paradigms, existing workload assessment techniques typically address only a single dimension of workload: They focus on either cognitive load induced by tasks such as arithmetic^21,22^, working memory^23^, stress^24^, or delays and interruptions in information flow^25^; or on physical load, reflected by spatial teleoperation performance^9^ or latency in sensory feedback^26^. On the other hand, in teleoperation, multitasking is expected under critical task conditions with little freedom to switch between the primary physical task (the teleoperation) and accompanying cognitive sub-tasks (decision making, coordination within the human team etc.). Consequently, it remains unclear which workload assessment techniques are suitable for accounting for both cognitive and physical workload elements and their interplay in teleoperation.

This article presents a controlled study to identify appropriate measures for real-time workload assessment in teleoperation with the ultimate goal of developing systematic robotic assistance. For this purpose, we designed an experiment, in which participants interact with a robotic teleoperation system to fulfill a physical ring-on-a-wire task. Meanwhile, they were presented with a secondary working memory task. We controlled the physical (teleoperation vs no teleoperation) and cognitive demand (as modulated by the secondary working memory task) to observe measurable differences in these two workload dimensions. We computed a variety of metrics using various physiological channels, including brain, cardiovascular and electrodermal activity, as well as robot movement and task performance data. Interestingly, we found that physiological measures can detect changes in either cognitive or physical workload individually, but not both simultaneously. Our findings suggest that robot performance data shows the most promise for developing a workload assessment system for teleoperation.

The article is organised as follows: The methodology described in detail in Section Methods. Section Results presents the experimental findings, followed by a discussion of these results and the study’s limitations in Section Discussion. Finally, Section Conclusion offers key insights and conclusions.

**Fig. 2:**
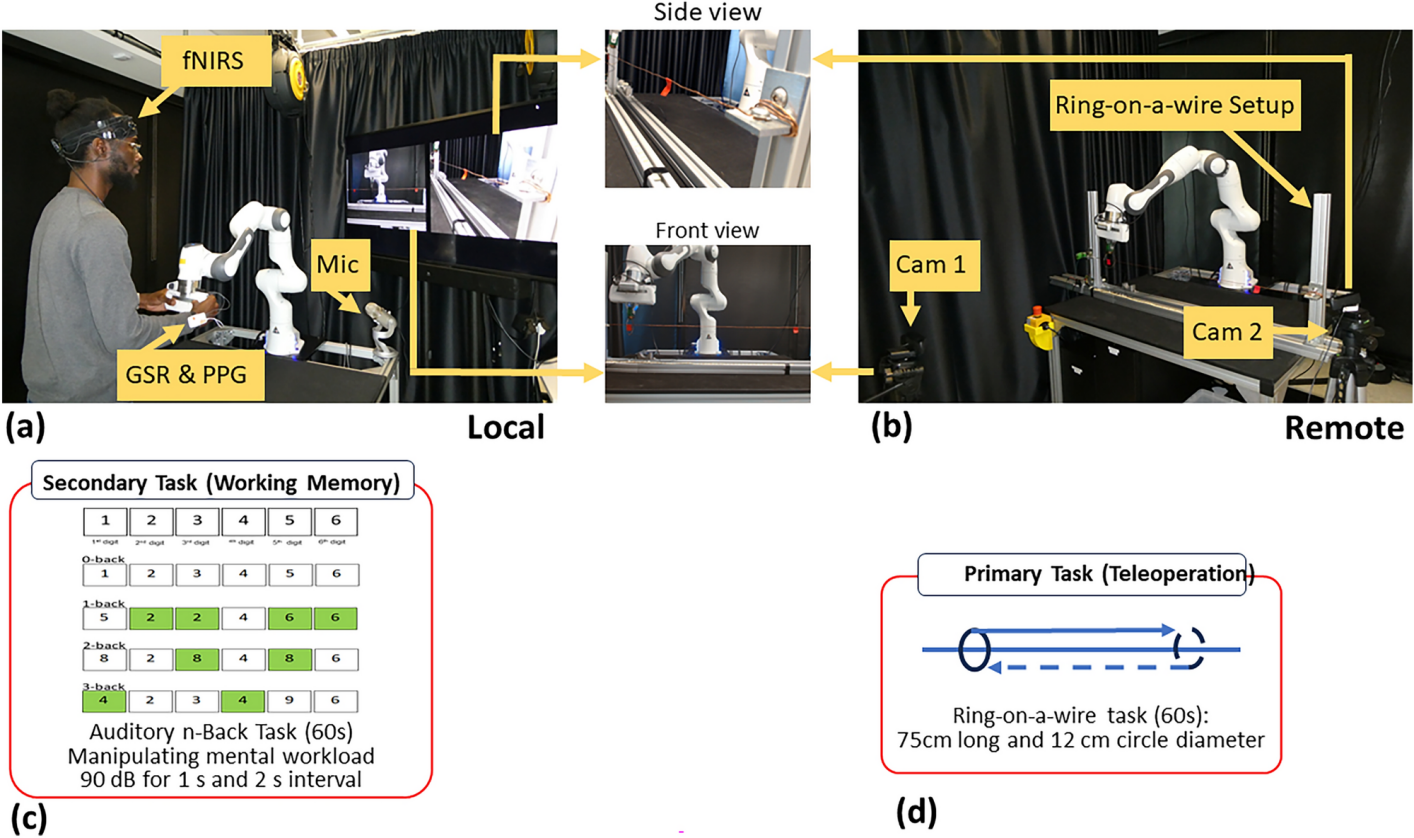
(**a**, **b**) The experimental setup uses a kinematically identical leader-follower teleoperation system. (**a**) The local arm (a.k.a. the leader) is controlled by the operator, while (**b**) the remote arm (a.k.a. the follower) is placed in a remote workplace. The operator moves the local arm and these movements are replicated by the remote arm. A participant engages in (**d**) a remote ring-on-wire task as the primary physical activity, concentrating on the visual display of front and side camera feeds while simultaneously performing (**c**) a secondary auditory n-back task to induce cognitive workload. In the subfigure n-back task is visually demonstrated for $n \in \{0,1,2,3\}$, where green squares are *hits*. The participant wears sensors to measure physiological data and a microphone records the participant’s responses to the secondary n-back task and subjective workload scores.

## Methods

### Robotic teleoperation setup

**Fig. 3:**
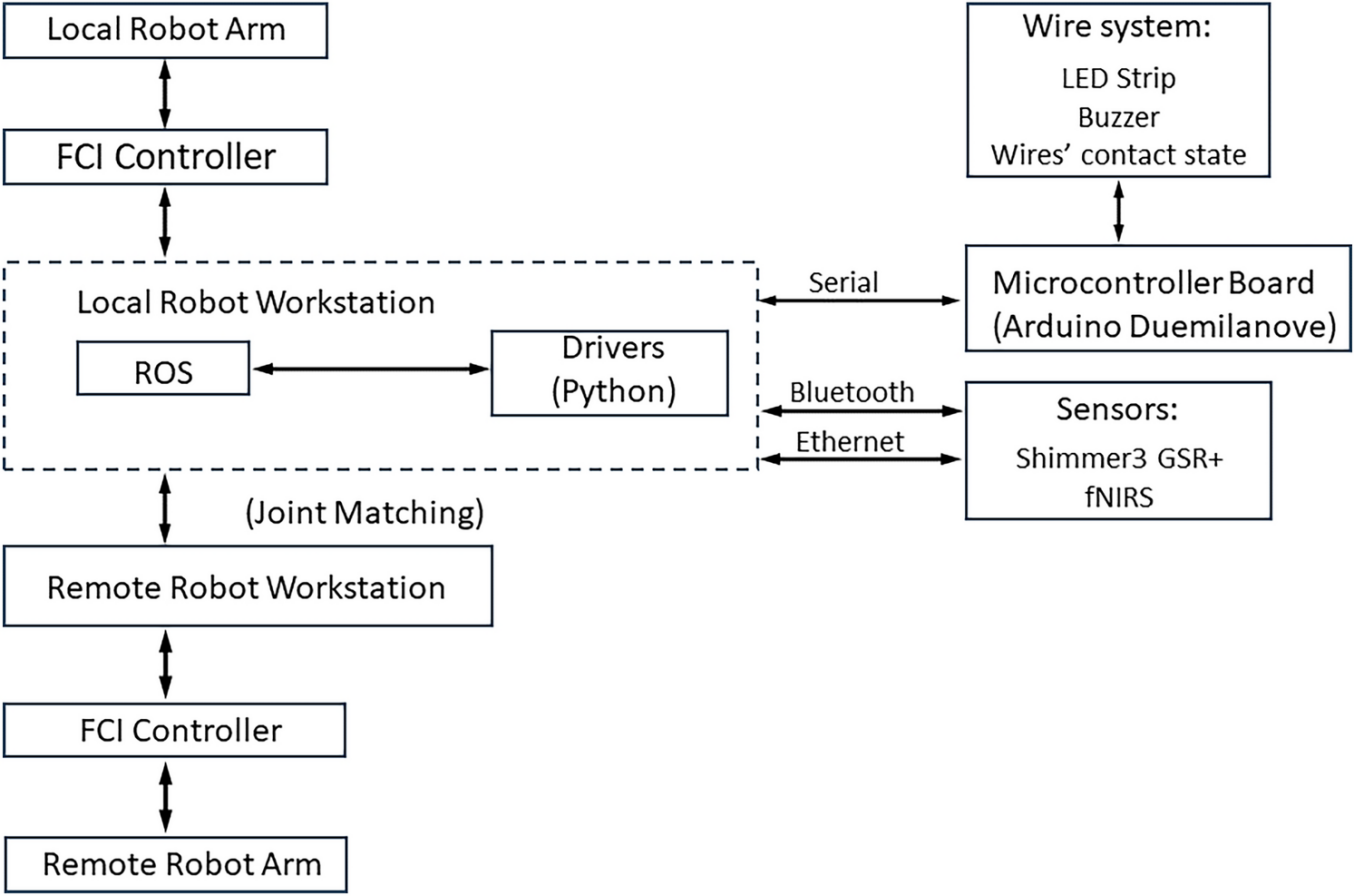
Teleoperation system architecture with workstations and sensor integration.

The teleoperation architecture is described in detail by Singh et al.^27^ and consists of two identical Franka Emika Panda robot arms (i.e. a local and a remote manipulator), arranged in a unilateral leader-follower setup as shown in Fig. 2. Each Panda arm is equipped with torque sensors integrated at joint actuators. A static Ethernet connection is utilised to link each robot to a dedicated workstation, minimising potential time delays. The Franka Control Interface (FCI) enables communication with the workstations through a low-level, bidirectional connection operating at 1 kHz. The open-source libfranka library is used to control the robots, providing various control options, including joint torques, positions and velocities, as well as Cartesian poses and velocities. Both the local and remote arms operate at a frequency of 1 kHz through the Simulation Lab (SL) robotics simulator and real-time control engine in real-time control mode. A control script running on the local system acts as a centralised point for controlling the robots. It also serves as a hub for ROS communication for integrating multiple sensors, such as the Octamon wireless fNIRS headset for brain activity monitoring and Shimmer GSR+ unit for GSR (Galvanic Skin Response) and PPG (Photoplethysmograph) measurements. It also incorporates an Arduino for real-time primary task performance monitoring, automatically tracking the contact between the ring and wire. This centralization simplifies operations and enhances coordinated data collection for accuracy, synchronization, and event tracking. The remote robot is connected to a dedicated controller and a separate remote PC with the capability of data exchange and coordination with the local robot workstation. This architecture is illustrated in Fig. 3. Custom Python scripts, using a wrapper-based approach and dedicated driver-based APIs, were implemented to ensure seamless integration of fNIRS and Shimmer sensors with the main ROS system, converting sensor data into ROS messages. The Arduino board is connected via rosserial, facilitating communication.

In our setup, the robotic arms are equipped with a zero torque controller for gravity compensation. This gravity compensation allows the operator to move the local arm effortlessly and intuitively through space as an extension of their movements without additional torque commands. The forward dynamics of an $n$-dof robot manipulator, where $n = 7$ in our setup, is given by (1), where $\mathbf{M}(\mathbf{q}) \in {\mathbb {R}}^{n \times n}$ represents the mass matrix for joint configuration, *q*, $\mathbf{C}(\mathbf{q}, \ddot{\mathbf{q}}) \in {\mathbb {R}}^{n \times n}$ accounts for the Coriolis effect and $\mathbf{g}(\mathbf{q}) \in {\mathbb {R}}^n$ denotes the joint torques due to gravity. In our setup, we omit *g*(*q*) because the robot arms are already gravity-compensated. The Coriolis term, $C(q,\ddot{q})$, is also negligible due to the low velocities of motion, hence omitted.

1 $$\begin{aligned} \tau = {M}(q) \ddot{q} + {C}(q, \dot{q}) \dot{q} + g(q) + \tau _{\text {ext}} \end{aligned}$$

where $q,\dot{q},\ddot{q} \in {\mathbb {R}}^n$ are the joint positions, velocities and accelerations, respectively and $\tau \in {\mathbb {R}}^n$ is the vector of joint actuation torques.

The remote robot acts on commands from the local robot and translates these instructions into physical actions by replicating the operator’s movements within the operational environment. The system uses a PD controller for unilateral position-position teleoperation to synchronise joints movements as formulated in (2):

2 $$\begin{aligned} \tau _{\text {cmd}} = K_p (q_r - q_l) - K_d \dot{q}_l , \end{aligned}$$

where $q_r$ and $q_l$ denote the remote and local joint angles, respectively. $K_p$ and $K_d$ are diagonal matrices that define the controller’s proportional and derivative gains, respectively, with set values: $K_p = diag([120,120,120,120,20,20,4])$ and $K_d = diag([10,10,10,10,6,5,3])$.

### Experimental tasks

#### Primary physical task: teleoperated ring-on-a-wire

The participants operated a local Franka Emika Panda arm to perform a ring-on-a-wire task with a kinematically identical remote arm, as illustrated in Fig. 2. The task required maneuvering a 12 cm diameter ring, attached to the gripper of the remote arm, along a straight wire spanning 75 cm between two designated points. Participants had 60 seconds to move the ring back and forth as many times as possible without touching the wire.

To prevent direct observation, the remote arm was concealed behind a curtain. Frontal and side views of the ring-on-wire setup were captured by two cameras (also shown in Fig. 2), and displayed side-by-side on a 65-inch screen, enabling participants to monitor and complete the remote task.

An Arduino board controlled both the buzzer and the LED strip. If the ring touched the wire at any point during the task, a circuit was completed that changed the voltage state of an Arduino’s input, activating a buzzer and an LED strip. Both the buzzer and the LED strip remained activated for as long as the contact was maintained. The Arduino communicated with the local arm computer via a serial port connection using the ROSSerial protocol, and published a ROS topic at 10 Hz to record the contact state between the ring and the wire.

#### Secondary cognitive task: auditory n-back working memory task

An auditory n-back task was implemented as a secondary task to modulate participants’ cognitive workload levels. The n-back task was chosen for its established efficacy in manipulating cognitive workload^28^. In the auditory n-back task, participants listened to a stream of spoken stimuli. The auditory stimuli were pre-recorded spoken digits ranging from 0 to 9, played through audio speakers at 90 dB for 1 second each, with a sampling rate of 16,000 Hz. The objective of the task was to determine whether each current stimuli matched the one heard ‘n’ steps ago in the sequence, by verbally responding “yes” for a match (hit) or “no” for a non-match (miss). For example, if ‘n’ is 2, participants compared the current digit with the digit heard two positions earlier. Following each digit presentation, there was a 2-second interval for participants to provide their verbal response. In the zero-back condition, participants were instructed to respond with a “yes” to every digit, serving as a baseline to control for brain activity related to speech production. Responses were recorded through a microphone, and analysed post-experiment to compute the n-back task accuracy.

Each n-back task lasted 60 seconds to match the duration of the teleoperation task, comprising 20 trials. Within these trials, 30% of the digits were designated as targets (digits matching the n-back criterion) and 70% as non-targets (digits not matching the criterion). The sequence of digits was presented in a pseudo-random order across all trials; hence, the auditory stimuli was uniform across participants, although an illusion of randomness was created per individual.

### Design and procedure

**Fig. 4:**
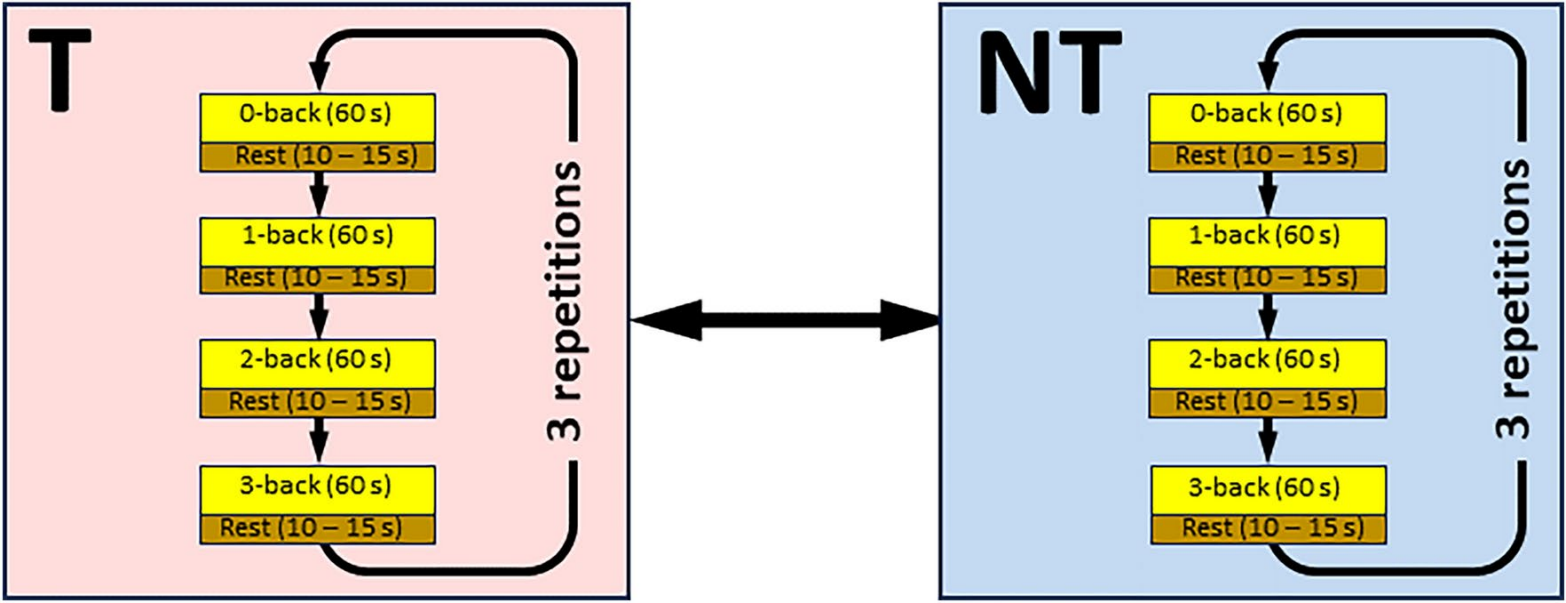
The experimental design features two blocks: Teleoperation [T] and No-Teleoperation [NT], which are presented to participants in counterbalanced order. Within each block, four task conditions (0-back, 1-back, 2-back, 3-back) were administered sequentially, however the starting condition for each participant was counterbalanced using a Latin square method. Each n-back trial lasted 60 seconds, with a 10–15 second rest interval between trials, during which participants responded to three subjective workload assessment questions. Each task block was repeated 3 times.

We controlled both the physical demand (Teleoperation [T] vs No Teleoperation [NT]) and cognitive demand (as varied by $n \in \{0,1,2,3\}$ in the auditory n-back task) to observe measurable differences in these workload dimensions. Participants engaged in two blocks: one in which they performed the teleoperation task and another, in which they stood with their hands on the robot workbench, maintaining an upright posture to prevent blood from rushing to the forehead. In each block, they completed three 60-second trials for each $n \in \{0,1,2,3\}$, with 10-15 second rest periods between trials. These trials coincided with the ring-on-a-wire task in the Teleoperation condition. During the rest periods between trials, participants verbally responded to three questions about perceived physical, cognitive, and overall workload, providing a subjective rating between 1 (lowest) to 5 (highest). The order of all conditions were counterbalanced using a Latin square method. Figure 4 illustrates the experimental blocks.

The study used a $2 \times 4$ within-subjects design. The independent variables were teleoperation mode (teleoperation, no-teleoperation) and n-back condition (0-back, 1-back, 2-back, 3-back). Participants took place in the experiment over two days, completing a familiarization session, followed by the experimental sessions.

#### Familiarization session

Participants attended a familiarization session at least one day prior to their experimental session to acquaint themselves with the tasks, mitigate novelty effects and enable more reliable brain data collection by controlling for brain plasticity-related responses. During this session, they practiced with both the n-back task and teleoperation until they felt comfortable with the procedures. No data were recorded during this session.

#### Experimental session

Upon arrival at the lab, participants received instructions regarding the experimental session and had the opportunity to ask questions. They then completed a brief demographic questionnaire consisting of 9 items, which took approximately 3 minutes. Following this, participants were fitted with the Octamon fNIRS headset and Shimmer sensors, and the researcher adjusted each fNIRS channel to ensure stable signal acquisition. Participants were then shown the teleoperation system once more and given a brief reminder about the ring-on-a-wire and n-back tasks.

Prior to the start of the experimental sessions, a three-minute resting period was recorded to establish a baseline of each participant’s brain activity using fNIRS. Following this baseline recording, the main data collection phase commenced.

During the experimental sessions, participants received brief prompts about the experimental conditions via prerecorded messages played over speakers. Each of the four n-back levels were performed in either the Teleoperation (T) condition, prompted by instruction “n-back, motion”, or the No-Teleoperation (NT) condition, prompted by instruction “n-back, no motion”, where n is a value between 0 and 3 depending on the current condition.

In T condition, participants were asked to actively observe the screen to move the local arm to execute the teleoperation, and simultaneously respond to the sequence of digits for the n-back task, by vocalising “yes” for a hit or “no” for a miss. At the end of the trial, participants heard a “Park” prompt, instructing them to guide the local arm back to the starting position on the wire, as marked by a red tape clearly visible in the camera feed. In NT, participants rested their arms on the workbench instead of performing the ring-on-a-wire task.

After each trial, regardless of the teleoperation condition, participants heard a “Rest” prompt. In T mode, this prompt meant they should let go of the local arm. In both modes, during rest, participants were asked to relax, and fix their gaze on a visual marker (a yellow sticker) attached to the local arm, while maintaining their arms in a restful position on the workstation. In this period, the experimenter asked participants to verbally rate their cognitive, physical, and overall workload. These concepts were explained to them prior to the beginning of the experiments. The total duration of the experiment was approximately 1 hour.

### Participants

Thirty-five healthy participants (22 males, 12 females, 1 non-binary, average age 25.9) took part in the experiment. Each participant signed an informed consent form, agreeing to the publication of identifying information and images in an online open-access publication. They received a £20 gift voucher as compensation for their time. Ethical approval was obtained from the School of Computer Science Ethics Committee of the University of Nottingham under reference CS-2021-R55 and all experiments were performed in accordance with relevant guidelines and regulations^29^.

### Metrics

In this study, we employ a variety of physiological and robotic performance measurements to evaluate physical and cognitive workload during robotic teleoperation. Additionally, we use a subjective workload assessment scale to gain insights into participants’ perceived physical, cognitive, and overall workload. This scale also serves to verify the effectiveness of the experimental conditions in altering the workload dimensions.

Performance measurements are indicators of robot teleoperation efficiency and effectiveness. The measured physiological data include electrodermal and cardiovascular measurements, as well as brain activity. Specifically, we utilise Galvanic Skin Response (GSR) and Photoplethysmography (PPG) to assess autonomic nervous system activity. Additionally, we measure functional Near-Infrared Spectroscopy (fNIRS) to monitor brain activity, providing valuable information on cerebral oxygenation and hemodynamics.

These measures, collectively, allow for a comprehensive analysis of both central and peripheral physiological responses, task performance, and subjective experiences, offering a holistic view of the body’s and mind’s reactions to changes in physical and cognitive workload levels.

#### Subjective perceptions of workload

We drew inspiration from the NASA Task Load Index (NASA-TLX) when selecting our subjective workload questions, which encompass both cognitive and physical aspects of workload. During piloting, we found that administering a NASA-TLX after each block was impractical for our methods: Participants had to leave the teleoperation workbench to complete paper or computer-based questionnaires, which would disrupt physiological data collection. Additionally, integrating NASA-TLX into the precise timing of tasks proved challenging, and overall participation duration became excessive. As a result, we opted for three single-dimensional rating scales:

- **Cognitive Workload:** How much mental and perceptual activity was required? Was the task easy or demanding, simple or complex?
- **Physical Workload:** How much physical activity was required? Was the task easy or demanding, slack or strenuous?
- **Overall Workload:** How hard did you have to work (mentally and physically) to accomplish your level of performance?

Each of these components was rated on a 5-pt Likert scale. The participants verbally reported their ratings after each trial when prompted. We presented participant with guidelines for interpretation as shown in Table 1.

**Table 1:** Participant instructions for the subjective workload scale.

Score	Workload	Spare Capacity
1	Under-utilised	Very high
2	Relaxed	Ample
3	Comfortable busy pace	Some
4	High	Very little
5	Excessive	None

#### Teleoperation performance measures

**N-back Accuracy:** Performance on the n-back task was quantified as the percentage of correctly identified responses.

**Number of Ring Touches:** The count of instances when the ring touches the wire, triggering the buzzer.

**Ring Contact Time:** The average duration of contact between the ring and the wire.

**Average Speed of the End Effector:** This metric quantifies the average speed of the remote arm’s end-effector throughout a trial. It indicates how quickly a participant operates the remote robot to complete the ring-on-a-wire task. The speed is calculated by dividing the total trajectory length covered during the trial by the trial’s duration.

3 $$\begin{aligned} {\text {Average speed}} = \frac{\sum _{i=1}^{N-1} \sqrt{(x_{i+1} - x_i)^2 + (y_{i+1} - y_i)^2 + (z_{i+1} - z_i)^2}}{t_{\text {finish}}({\mathrm{trial}}) - t_{\text {start}}({\mathrm{trial}})} , \end{aligned}$$

where *N* is the number of data samples in a trial; $x_i$, $y_i$ and $z_i$ are respectively the *x*, *y* and *z* Cartesian coordinates of the remote arm’s end effector for the *i*th sample; and $t_{\mathrm {start}}(\text {trial})$ and $t_{\mathrm {finish}}(\text {trial})$ are respectively start and end times of the trial.

**Average Power Consumption:** This metric represents the robot arm’s average power output, providing a measure of the participant’s efficiency in controlling the robot arm. The average power is calculated as:

4 $$\begin{aligned} \text {P} = \frac{1}{N} \sum _{i=1}^{N} \left( \sum _{j=1}^{7} |\tau _{i,j} \cdot \dot{\theta }_{i,j}| \right) , \end{aligned}$$

where $\tau _{i,j}$ is the torque applied at the *j*th joint at the *i*th time point. $\dot{\theta }_{i,j}$ is the angular velocity of the *j*th joint at the *i*th time point.

**Total Energy Consumption:** This metric quantifies the total energy expended by the robot arm during a trial, reflecting the overall energy demands placed on the robot and the participant controlling it. It takes into account both the intensity and duration of the interaction. Total energy consumed is computed as:

5 $$\begin{aligned} E = \sum _{i=1}^{N} \left( \sum _{j=1}^{7} |\tau _{i,j} \cdot \dot{\theta }_{i,j}| \right) \Delta t_i , \end{aligned}$$

where $\tau _{i,j}$ is the torque applied at the *j*th joint at the *i*th time point, $\dot{\theta }_{i,j}$ is the angular velocity of the *j*th joint at the *i*th time point, and $\Delta t_i$ is the duration between *i*th and $i-1$th samples.

**Robot Motion Smoothness:** Spectral arc length (SAL)^30^ is computed using the local robot’s end-effector speed for each trial to quantify motion smoothness. SAL reflects the participant’s ability to execute fluid and controlled movements, which is crucial for precision and efficiency in robotic manipulation tasks. It is derived by computing the SAL of the frequency spectra of the speed profiles of the end-effector of the local arm during a trial. SAL is typically a negative metric when computed using speed profiles, and values closer to zero indicate smoother profiles.

#### Electrodermal and cardiovascular measurements

**Galvanic skin response (GSR)** is also known as skin conductance or electrodermal activity (EDA), and is measured using exosomatic methods that pass a small current, either AC or DC, through the skin to determine its resistance^31,32^. GSR is significantly influenced by eccrine sweat glands^31^, which are regulated by the sympathetic nervous system. When a person is aroused, sweat gland activity increases, leading to higher skin conductance. These glands are linked to psychological responses and stimulus processing, unlike other glands primarily involved in temperature regulation^32^.

We used a Shimmer3 GSR+ device to record GSR data. This device uses reusable electrodes attached to two fingers on one hand to measure the electrical conductance. The GSR data were acquired at 10 Hz. A third-order low-pass filter with a cutoff frequency of 1Hz was applied to the GSR signal to remove motion artefacts^33^. Since GSR signals vary significantly between individuals, we normalised each participant’s data using mean normalisation^34^.

We use the **average normalised GSR** as a workload measure. This metric is calculated by subtracting the mean GSR value of each trial from the raw time series data for each participant and then averaging the result:

6 $$\begin{aligned} \text {average GSR} = \frac{1}{N} \sum _{i=1}^{N} (\text {GSR}_i - \frac{1}{N} \sum _{i=1}^{N} \text {GSR}_i) , \end{aligned}$$

where *N* is the number of data samples in a trial.

**Cardiovascular response** is expected to be significantly impacted by increasing workload^35^. Elevated workload typically results in heightened sympathetic activity and diminished parasympathetic activity^36^. Under increased cognitive load, the sympathetic nervous system response is expected to lead to increased heart rate and decreased heart rate variability.

Shimmer3 GSR+ device was also used to measure Photoplethysmography (PPG) data by attaching an optical pulse probe to a finger. The AC component of the PPG signal indicates the heart’s rhythmic blood volume changes. PPG also efficiently measures Pulse Rate Variability (PRV) based on the time intervals between adjacent cardiac cycles^37,38^. The PPG data were acquired at 10 Hz. We extracted the following metrics using the PPG data:

**Pulse Rate:** Pulse rate is the rate at which the arteries expand and contract in response to heartbeats due to blood volume changes in the microvascular bed of tissue. It is closely related to the heart rate and reflects the same physiological process^39,40^. The pulse rate is computed as the ratio of 60 seconds to the time interval between consecutive PPG peaks. The average pulse rate is computed as the mean of these individual pulse rate values over a given period:

7 $$\begin{aligned} PR = \frac{1}{P-1} \sum _{i=1}^{P-1} \left( \frac{60}{\Delta t_i} \right) , \end{aligned}$$

Where *P* is the number of PPG peaks in a trial, and $\Delta t_i$ is the time duration between two consecutive peaks in a trial, also known as the interbeat interval (IBI).

**Pulse Rate Variability:** Closely related to heart rate variability, pulse rate variability refers to the variation in time intervals between consecutive beats as measured by changes in blood volume. This metric indicates the heart’s adaptability and the overall balance of the autonomic nervous system. It is obtained by measuring the time intervals between consecutive peaks in PPG data during a trial. In this study, the average pulse rate variability is computed as:

8 $$\begin{aligned} PRV = \frac{1}{P-1} \sum _{i=1}^{P-1} \Delta t_{i} , \end{aligned}$$

Where *P* is the number of PPG peaks in a trial, and $\Delta t_i$ is the time duration between two consecutive peaks in a trial.

### Brain activity measurements

The assessment of brain activity has become increasingly popular as a physiological measure of workload, based on the idea that task-related brain activity consumes specific mental resources, correlating with the cognitive complexity of a task^41^. Functional near-infrared spectroscopy (fNIRS) offers portable, movement-friendly imaging that effectively measures workload through variations in brain hemoglobin levels in response to varying working memory loads^23^. Unlike fMRI and Positron Emission Tomography (PET), which rely on neurovascular coupling^42,43^, or EEG and Magnetoencephalography (MEG), which are based on electromagnetic brain activity^44,45^, fNIRS offers robust monitoring of cortical hemodynamics with minimal sensitivity to bodily movements^46^. fNIRS is thus beneficial in investigating workplace tasks, providing insights into areas for improvement through enhanced interfaces or systematic assistance.

**Fig. 5:**
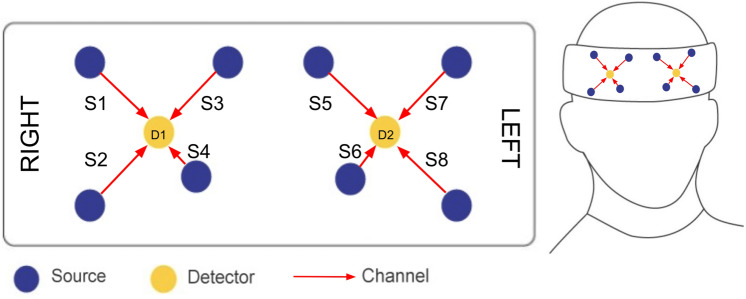
Diagram showing the location of the emitters (S1-S8) and receivers (D1 and D2) in the fNIRS probe. HbO and HbR are calculated for each channel, which is made up of the eight emitter-receiver pairs. S4 and S6 are short-separation channels.

In this study, we measure brain activity using the Octamon wireless fNIRS system (Artinis Medical Systems, Elst) and the Oxysoft software (version 3.2.72). The fNIRS probe covers the frontal cortex (over the forehead) where mental workload is typically observed, as shown in Fig. 5. The octamon has 8 infrared light emitters and 2 detectors, creating 8 distinct data channels, including two short-separation channels (S4 and S6 as shown in Fig. 5), which were all acquired at 10 Hz. The distance between emitters and detectors in data channels is 35 mm and measures activity in the brain. The short-separation channels (S4 and S6), at 10 mm, measure blood oxygenation in the skin, in order to subtract this data as noise. fNIRS emitters use 760 nm and 850 nm wavelengths, which reflect oxygenated and deoxygenated hemoglobin, respectively:

- **Oxygenated Hemoglobin (HbO)** is the amount of blood cells containing oxygen, indicating activation in the brain.
- **Deoxygenated Hemoglobin (HbR)** is the amount of cells where oxygen has been used. HbR should change inversely to HbO in order for HbO changes to be considered as workload changes.

Brain activation leads to an increase in HbO in order to function, and in tandem this should lead to a decrease in HbR. Therefore, only channels with a negatively correlated haemoglobin species (an increased HbO and decreased HbR at $q < 0.5$) are considered significant^47^. An increase or decrease in both HbO and HbR at the same time, or a change in only one of the variables is considered a noise artefact.

The analysis of the fNIRS data was carried out using the NIRS Toolbox^48^. Initially, the raw data were downsampled to a frequency of 4Hz to improve the signal-to-noise ratio^49^. The raw signals were then converted into optical density changes and subsequently into measures of oxygenated (HbO) and deoxygenated hemoglobin (HbR) using the Beer-Lambert law, incorporating a partial path length correction factor of 0.1 for both wavelengths^50^. To mitigate the impact of motion artifacts, the Temporal Derivative Distribution Repair (TDDR) method was applied. Additionally, a short-separation channel was included as a regressor in the General Linear Model (GLM) to correct for physiological noise^51^. Beta coefficients, indicative of activations, were determined using the autoregressive iteratively reweighted least squares method^52^. Finally, the hemodynamic response was modeled using the BoxCar function.

## Results

### Subjective perceptions of workload

**Fig. 6:**
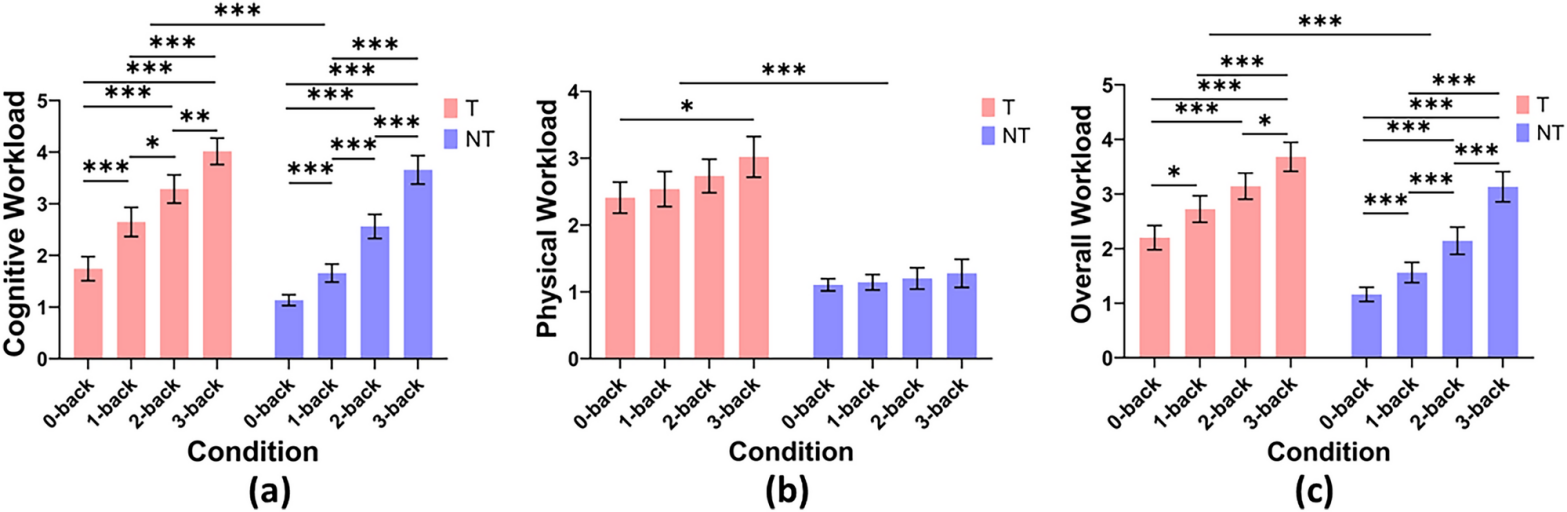
Subjective workload ratings across conditions. (**a**) Perceived cognitive workload (**b**) perceived physical workload (**c**) perceived overall workload. Asterisks (*) over bars indicate statistically significant differences with *$p<0.05$, **$p<0.01$, ***$p<0.001$. Error bars use 95% CI.

Figure 6 shows the subjective workload ratings. These Likert scale data were analysed using non-parametric tests due to the ordinal nature of the responses. Specifically, the Wilcoxon signed-rank paired test was used for comparisons between T and NT, and the Kruskal–Wallis was used for discovering main effects of the n-back condition. In case of main effects, Dwass-Steel-Crichtlow-Fligner (DSCF) non-parametric post-hoc tests were used for pairwise comparisons between n-back levels. As DSCF is designed to control the familywise error rate inherently, we do not perform additional alpha correction for multiple comparisons.

#### Perceived cognitive workload

A Wilcoxon signed-rank test revealed a significant difference in perceived cognitive workload between the T and NT conditions ($z = -8.263, p < 0.001$). The Ranks table showed that there were 13 negative ranks ($N = 13, {\text {Mean Rank}} = 28.00, \text {Sum of Ranks} = 364.00$), 101 positive ranks ($N = 101, {\text {Mean Rank}} = 61.30, \text {Sum of Ranks} = 6191.00$), and 26 ties ($N = 26$), demonstrating that the perceived cognitive workload ratings were generally higher under the teleoperation condition compared to the no teleoperation condition. This suggests that participants perceived teleoperation as imposing additional cognitive demands rather than solely physical ones.

Kruskal–Wallis tests revealed significant differences in perceived cognitive workload between the different levels of the n-back task under NT ($\chi ^2(3) = 107, p < 0.001, \varepsilon ^2 = 0.750$) and under T ($\chi ^2(3) = 80.0, p < 0.001, \varepsilon ^2 = 0.560$). Pairwise post-hoc comparisons indicated significant differences between all levels of the n-back task as summarised in Table 2, with lower n-back levels consistently corresponding to lower perceived cognitive workload.

These results confirm that the n-back task was able to manipulate the perceived cognitive workload as expected under the NT condition and observed significant differences were maintained whilst performing teleoperation in T.

**Table 2:** Pairwise comparisons of perceived cognitive workload between n-back conditions under NT and T.

		NT Condition		T Condition	
		W Value	*p* value	W Value	*p* value
0-back vs.	1-back	7.04	< 0.001	6.20	< 0.001
	2-back	10.16	< 0.001	8.95	< 0.001
	3-back	10.65	< 0.001	9.96	< 0.001
1-back vs.	2-back	7.58	< 0.001	4.36	0.011
	3-back	9.75	< 0.001	8.11	< 0.001
2-back vs.	3-back	7.62	< 0.001	5.17	0.001

#### Perceived physical workload

A Wilcoxon signed-rank test revealed a significant difference in perceived physical workload between the T and NT conditions ($z = -9.988, p < 0.001$). The Ranks table showed that there were 0 negative ranks ($N = 0$), indicating that no perceived physical workload ratings under the teleoperation condition were lower than the perceived physical workload ratings under the no teleoperation condition. However, there were 132 positive ranks ($N = 132, {\text {Mean Rank}} = 66.50, \text {Sum of Ranks} = 8778.00$) and 8 ties ($N = 8$), indicating that perceived physical workload ratings were generally higher under the teleoperation condition compared to the no teleoperation condition. This suggests that physical workload was significantly greater during teleoperation, as expected

The Kruskal–Wallis test revealed no significant differences in perceived physical workload under NT across the different levels of the n-back task ($\chi ^2(3) = 0.590, p = 0.899, \varepsilon ^2 = 0.0004$). This indicates that participants did not experience significantly different levels of physical workload during n-back tasks alone.

On the other hand, under T, the Kruskal–Wallis test revealed significant differences in physical workload across the different levels of the n-back task ($\chi ^2(3) = 11.2, p = 0.011, \varepsilon ^2 = 0.007$). Pairwise comparisons indicated that physical workload was perceived to be significantly lower in the 0-back condition compared to the 3-back condition ($W = 4.15, p = 0.018$), but other comparisons were not significant. The results are presented in Table 3.

**Table 3:** Pairwise comparisons of perceived physical workload between n-back conditions under NT and T.

		NT Condition		T Condition	
		W Value	*p* value	W Value	*p* value
0-back vs.	1-back	-0.238	0.998	1.40	0.755
	2-back	0.618	0.972	3.33	0.086
	3-back	0.708	0.959	4.15	0.018
1-back vs.	2-back	0.784	0.945	1.78	0.589
	3-back	0.888	0.923	2.97	0.152
2-back vs.	3-back	0.153	1.000	1.63	0.657

#### Perceived overall workload

A Wilcoxon signed-rank test revealed a significant difference in perceived overall workload between the T and NT conditions ($z = -9.372, p < 0.001$). The Ranks table showed that there were 6 negative ranks ($N = 6, {\text {Mean Rank}} = 27.50, \text {Sum of Ranks} = 165.00$), 120 positive ranks ($N = 120, {\text {Mean Rank}} = 65.30, \text {Sum of Ranks} = 7836.00$), and 14 ties ($N = 14$), demonstrating that the perceived overall workload ratings were generally higher under the teleoperation condition compared to the no teleoperation condition. This suggests that the overall workload was significantly higher while performing teleoperation.

The Kruskal–Wallis test revealed significant differences in overall workload across the different levels of the n-back task under NT ($\chi ^2(3) = 85.2, p < 0.001, \varepsilon ^2 = 0.596$) and T ($\chi ^2(3) = 53.1, p < 0.001, \varepsilon ^2 = 0.372).$ Pairwise comparisons indicated significant differences between all levels of the n-back task, except between 1-back and 2-back in T, as summarised in Table 4. Lower n-back levels consistently corresponded to lower perceived cognitive workload. These findings suggest that perceptions of overall workload increases consistently with the difficulty of the n-back tasks. This is a trend that primarily echoes the results of the cognitive workload component rather than the experienced physical workload.

**Table 4:** Pairwise comparisons of perceived overall workload between n-back conditions under NT and T.

		NT condition		T condition	
		W Value	*p* value	W Value	*p* value
0-back vs.	1-back	5.54	< 0.001	4.34	0.011
	2-back	8.31	< 0.001	6.87	< 0.001
	3-back	10.23	< 0.001	8.85	< 0.001
1-back vs.	2-back	5.38	< 0.001	3.42	0.074
	3-back	9.12	< 0.001	6.50	< 0.001
2-back vs.	3-back	7.01	< 0.001	3.93	0.028

### N-back task performance

**Fig. 7:**
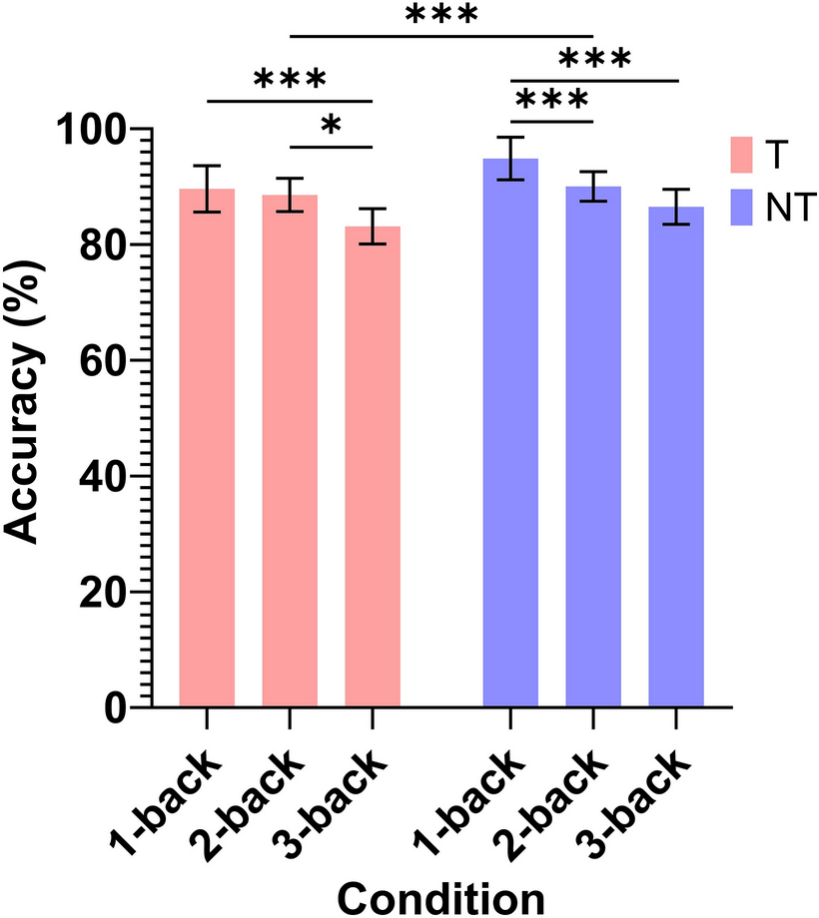
Accuracy of n-back task under T and NT conditions for each n-back level. Asterisks between bars indicate statistically significant differences between conditions with *$p<0.05$, ***$p<0.001$. Error bars indicate the standard error of the mean (SEM).

Figure 7 shows the mean accuracy of the n-back task for all conditions, which is the percentage of correctly identified responses. A Shapiro-Wilks test revealed that the data were not normally distributed $(W = 0.683,p <0.001),$ so the n-back task performance was analysed using non-parametric tests. Specifically, the Wilcoxon signed-rank paired test was used for comparisons between T and NT, and the Kruskal–Wallis was used for discovering main effects of the n-back condition. In case of main effects, DSCF was used for pairwise comparisons between n-back levels.

A Wilcoxon signed-rank test revealed a significant difference in average accuracy between the NT and T conditions ($z = -4.394, p < 0.001$). The Ranks table showed that there were 27 negative ranks ($N = 27, {\text {Mean Rank}} = 37.57, \text {Sum of Ranks} = 1014.50$), 65 positive ranks ($N = 65, {\text {Mean Rank}} = 50.21, \text {Sum of Ranks} = 3263.50$), and 13 ties ($N = 13$), demonstrating that n-back performance was generally higher under the no teleoperation condition compared to the teleoperation condition. The results indicates that performing a teleoperation task simultaneously as the n-back task reduced the n-back performance.

Consistent with findings in the literature for n-back tasks performed without additional stimuli^53,54^, Kruskal–Wallis tests revealed significant differences in accuracy across the different levels of the n-back task under both NT ($\chi ^2(3) = 106, p < 0.001, \varepsilon ^2 = 0.763$) and T conditions ($\chi ^2(3) = 89.3, p < 0.001, \varepsilon ^2 = 0.642$).

Pairwise comparisons showed that under NT, accuracy was significantly better in the 1-back condition compared to the 2-back ($W = -8.04, p < 0.001$) and the 3-back ($W = -8.17, p < 0.001$) conditions. Under T, accuracy was significantly higher in the 1-back condition compared to the 3-back condition ($W = -5.57, p < 0.001$) and higher in the 2-back condition compared to the 3-back condition ($W = -3.96, p = 0.026$).

### Teleoperation performance

**Fig. 8:**
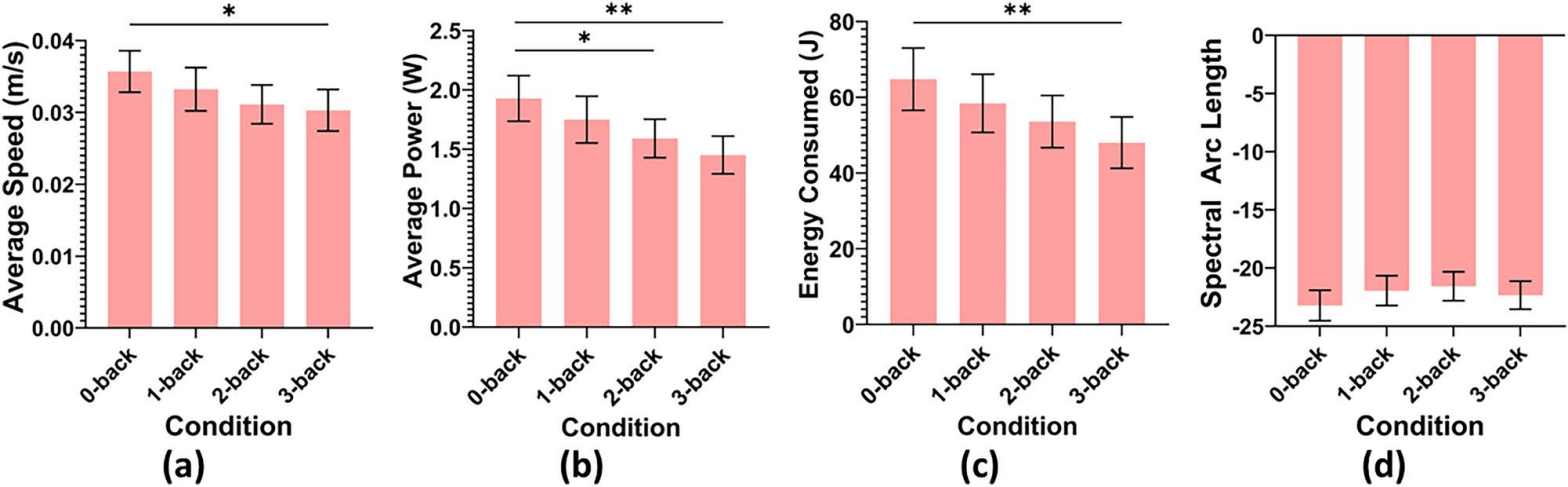
Robot performance metrics across the different workload conditions under T. (**a**) Average speed (**b**) Average power (**c**) Energy consumption (**d**) Average robot joint motion smoothness (spectral arc length^30^). Asterisks (*) over bars indicate statistically significant differences between conditions with *$p<0.05$, **$p<0.01$. Error bars use 95% CI.

Several metrics are utilised to represent teleoperation performance, as detailed in “Section Metrics”. Since robot performance data exist only in the T condition, analyses were conducted using one-way ANOVAs, followed by Tukey posthoc multiple comparison tests. ANOVA showed no significant differences in the number of ring touches or the ring contact time between the four n-back conditions. The mean of other performance measures are illustrated in Fig. 8.

**Average Speed of the End Effector** ANOVA indicated significant differences in average speed of the remote robot’s end effector ($F(3,416) = 2.761, p = 0.042$). Post-hoc analysis using the Tukey test demonstrated that the average speed in the 0-back condition ($0.0357 \pm 0.0149 m/s$) was significantly higher than in the 3-back condition ($0.0303 \pm 0.0149 m/s, p = 0.044$). No other comparisons between n-back conditions showed significant differences in average speed.

**Average Power Consumption** ANOVA revealed significant differences between n-back conditions in average power consumption ($F(3,416) = 5.222, p = 0.002$). The Tukey post-hoc test indicated that the average power was significantly lower in the 2-back ($1.59 \pm 0.84 W, p = 0.041$) and 3-back conditions ($1.45 \pm 0.82 W, p = 0.001$) compared to the 0-back condition ($1.93 \pm 0.99 W$). There were no significant differences found in comparisons between other workload conditions.

**Total Energy Consumption** ANOVA also showed significant differences in the total energy consumption between workload conditions ($F(3,416) = 3.622, p = 0.013$). Post-hoc Tukey tests revealed that energy consumed in the 0-back condition ($64.79 \pm 42.51 J$) was significantly higher than in the 3-back condition ($48.03 \pm 35.03 J, p = 0.009$). No significant differences in energy consumption were observed between the other workload conditions.

**Robot Motion Smoothness** ANOVA for robot motion smoothness across different n-back conditions revealed no significant differences ($F(3,416) = 1.251, p = 0.291$).

### Electrodermal and cardiovascular measurements

**Fig. 9:**
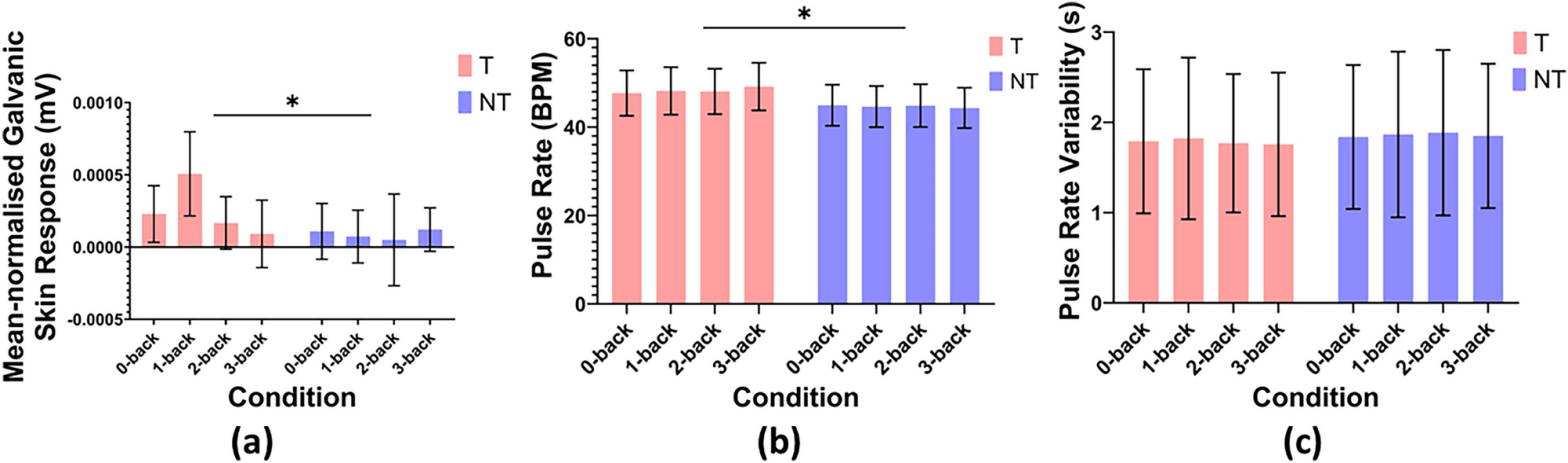
GSR and PPG relationships between teleoperation and working memory conditions. (**a**) Mean-normalised GSR indicating skin conductance response (**b**) The PPG-derived pulse rate (PR) reflecting blood volume changes at the finger (**c**) The PPG-derived pulse rate variability (PRV) indicating the inter-beat interval. Asterisks (*) over bars indicate statistically significant differences with $p<0.05$. Error bars use 95% CI.

Galvanic skin response (GSR) and Photoplethysmography (PPG) data were recorded from 34 participants out of 35, as data recording failed for one individual. Mean normalised GSR, heart rate and heart rate variability are computed from the recorded data as mentioned in “Section Electrodermal and cardiovascular measurements”. Figure 9 illustrates the means and confidence intervals for these physiological variables for the teleoperation conditions across all levels of the n-back task.

#### Galvanic skin response (GSR)

A two-way ANOVA revealed a significant main effect of the teleoperation condition on the mean-normalised GSR ($F(1, 800) = 3.993, p = 0.046$), with a very small effect size (partial $\eta ^2 = 0.005$). Specifically, GSR was higher in T, compared to NT. The main effect of the n-back conditions was not significant ($F(3, 800) = 1.148, p = 0.329,$ partial $\eta ^2 = 0.004$). No interaction effects were found ($F(3, 800) = 1.484, p = 0.217$, partial $\eta ^2 = 0.006)$.

#### Pulse rate (PR)

A two-way ANOVA revealed that the main effect of teleoperation was statistically significant ($F(1, 800) = 4.073, p = 0.044$, partial $\eta ^2 = 0.005$), suggesting that teleoperation has an effect on pulse rate, albeit with a small effect size. The main effect of the n-back condition on pulse rate was not significant ($F(3, 800) = 0.011, p = 0.998$, partial $\eta ^2 = 0.000$), suggesting that an increasing cognitive demand does not significantly influence pulse rate, although physical demand does. No significant interaction effects were found ($F(3, 800) = 0.063, p = 0.979$, partial $\eta ^2 = 0.000$).

#### Pulse rate variability (PRV)

A two-way ANOVA analysis revealed that neither the main effect of teleoperation ($F(1, 800) = 1.656, p = 0.199,$ Partial $\eta ^2 = 0.002$) nor the main effect of the working memory conditions ($F(3, 800) = 0.090, p = 0.966,$ Partial $\eta ^2 < .000$) on PRV were statistically significant. This suggests that neither teleoperation or working memory conditions alone impacted the PRV. No significant interaction effects were found $(F(3, 800) = 0.090, p = 0.965,$ Partial $\eta ^2 = 0.000)$.

### Brain activity measurements

To analyse fNIRS data, we employed a General Linear Model (GLM) approach. This method allows for the subtraction of data from the short-distance channels and considers the shape of the fNIRS signal rather than its mean values. After performing the GLM analysis, we applied a mixed-effects model to each channel independently to assess the impact of each experimental condition as fixed effects, with participants treated as random effects. This model helps identify significant changes in oxyhemoglobin (HbO) and deoxyhemoglobin (HbR) levels, attributing these changes to the independent variables for each fNIRS channel. Shifts in blood oxygen levels show brain activation as an impact of mental workload. Specifically, an activation due to an increase in mental workload is characterised by an increase in HbO and a decrease in HbR. For multiple comparisons, we utilised the false discovery rate (FDR) correction, setting the significance threshold at 0.05 ($q \le 0.05$) as per Benjamini and Hochberg’s method^55^. Finally, we conducted contrast analyses to evaluate differences between experimental conditions. For any significant differences found, we verified that the changes were present in opposite directions for both HbO and HbR to ensure that the observed changes were due to neural activation rather than artefacts, such as those caused by motion.

We observed a significant HbR decrease in channel S5-D2, located over the left dorsolateral prefrontal cortex (DLPFC), in response to the teleoperation variable irrespective of the n-back condition ($beta = 1.86, SE = 3.80, t = -7.09, q < 0.001, power = 0.96$). However, there was no corresponding significant increase in HbO. The mixed effects model identified a significant difference over channel S5-D2 between T and NT, while performing the 1-back task.

**Fig. 10:**
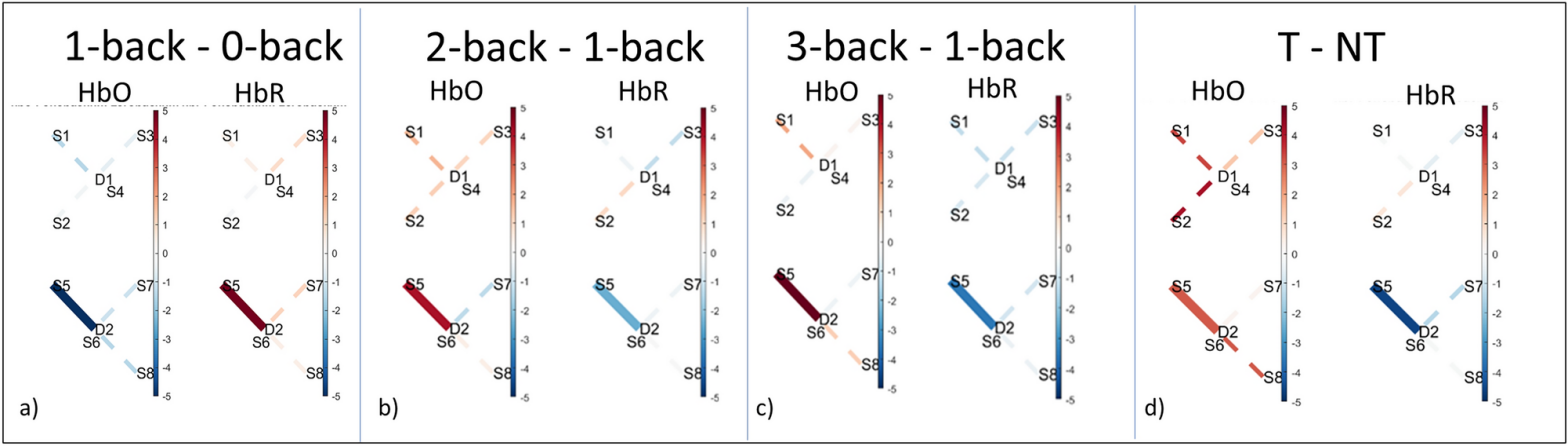
Results of contrast analysis from fNIRS data. Solid bold lines indicate significant channels (source-detector pairs) where differences in HbO and HbR concentrations were detected. Dashed lines are not significant. Results are only shown if HbO and HbR exhibit significant changes in the expected opposite directions. Instances where only one of these measures changes are considered noise artifacts. The left panel shows differences in NT across different tasks: (**a**) 1-back–0-back, (**b**) 2-back–1-back, (**c**) 3-back–1-back. (**d**) presents significant findings from the 1-back task, comparing T vs NT. Significance in contrasts is represented through a colour scale reflecting t-statistics, with the intensity of red and blue colours indicating the magnitude of HbO and HbR changes, respectively. Red and blue respectively represent positive and negative t-statistics values.

As shown in Fig. 10, we observed a significant increase in HbO levels ($beta = 3.10, SE = 0.99, t = 3.10, q = 0.012, power = 0.90$) and a significant decrease in HbR levels ($beta = -4.60, SE = 0.93, t-statistic = -4.90, q < 0.001, power = 0.96$). We also observed a significant increase in HbO in channel S8-D2, which is also over the left DLPFC, in the 0-back task under T condition compared to NT ($beta = 6.39, SE = 2.12, t = 3.00, q = 0.035, power = 0.42$). However, contrary to normal expectations, this increase in HbO was not accompanied by a corresponding decrease in HbR, meaning that it cannot be confirmed as significant.

The mixed effects model did not identify a consistent significant effect of n-back conditions on brain activity. Under the 1-back condition, we observe a significant decrease in HbR in S8-D2 $(beta = -1.62, SE = 0.47,t = -3.45, q = 0.23, power = 0.76).$ Similarly, under the 3-back condition, a significant decrease in HbR was observed in S8-D2 ($beta = -1.98, SE = 0.47,t = -4.21, q = 0.02, power = 0.76$). However, neither of these were associated with a significant in HbO regardless of whether participants were performing teleoperation or not.

Under NT, comparing the 1-back task to the 0-back task, our analysis found a significant decrease in HbO ($beta = -5.50, SE = 0.99, t = -5.51, q < 0.001, power = 0.89$) and a significant increase in HbR ($beta = 4.53, SE = 0.93, t = 4.84, q < 0.001, power = 0.95$) in the left DLPFC, this time in channel S5-D2. We note here that this response is contrary to what is normally expected. More in line with what is expected, there was a significant increase in HbO $(beta = 4.13, SE = 0.99, t = 4.16, q < 0.001, power = 0.90)$ and a decrease in HbR ($beta = -2.51, SE = 0.93, t = -2.69, q = 0.04, power = 0.96$) in the same channel, when comparing the 2-back task to the 1-back task. Similarly, we observed a significant increase in HbO ($beta = 5.13, SE = 0.99, t = 5.15, q < 0.001, power = 0.90$) and a decrease in HbR ($beta = -3.56, SE = 0.93, t = -3.80, q < 0.001, power = 0.95$) in the S5-D2 during the 3-back task in contrast to the 1-back task.

Under T, the mixed effects model did not find the same significant differences. The only significant difference found was between the 0-back and 1-back conditions: there was a noticeable increase in HbO ($beta = 2.50, SE = 0.99, t = 2.51, q = 0.007, power = 0.90$); however, there was no corresponding decrease in HbR, indicating a potential noise. This implies that the significant differences observable by fNIRS performing different n-back tasks alone were no longer observable for the workload induced by the teleoperation task.

## Discussion

This study evaluated the effectiveness of various metrics in assessing workload as a combination of physical and cognitive demands during robotic teleoperation tasks. These metrics included teleoperation performance, physiological data, and subjective workload scores. Notably, the number of ring touches and the error duration did not vary significantly across conditions, suggesting that participants may have adapted their strategies to manage workload changes while maintaining accuracy in avoiding ring-wire contact. In the rest of this section, we discuss the results and highlighting key findings, study limitations, and directions for future work.

### How workload was experienced during teleoperation

Performance measures and subjective ratings confirmed that workload was successfully manipulated by the primary teleoperation task and the secondary n-back task. Comparative analysis between Teleoperation (T) and No-Teleoperation (NT) conditions revealed that teleoperation adds a layer of workload, reducing n-back task accuracy while increasing perceived cognitive, physical, and overall workload. In NT, participants performed better on lower-complexity n-back tasks (1-back) compared to higher complexity ones (3-back). This performance difference persisted during teleoperation in the T condition, consistent with previous studies using the n-back task^53,54^.

Subjective workload perceptions varied significantly across cognitive, physical, and overall workload variables under the teleoperation and n-back conditions. Perceived cognitive workload increased with n-back task difficulty, and these effects were amplified under teleoperation, indicating an extra cognitive load beyond the n-back task alone. Perceived physical workload was also significantly higher in T, compared to NT, particularly in the most challenging n-back condition (3-back), suggesting that physical demands are heightened when cognitive workload is high. This perhaps indicates that physical workload during teleoperation is exacerbated by high cognitive workload. Overall workload trends were influenced by both cognitive and physical workload components, indicating that participants’ general workload experience during teleoperation is not determined solely by one factor.

These findings highlight that secondary task complexity and the existence of teleoperation significantly affect the perceived workload. The comparison between tasks done with and without teleoperation underscores the extra cognitive and physical effort required for teleoperation, emphasising the importance of understanding the impact of secondary tasks on operators.

### Measuring workload during teleoperation for dynamic task demand

While subjective workload scores and task performance varied with n-back task difficulty during teleoperation, objective physiological measures did not show discernible differences across these levels. GSR and PR variables significantly differed between T and NT conditions, yet remained unaffected by different cognitive workload levels imposed by the n-back tasks. While physiological measures like GSR and PR indicated significant responses to teleoperation, the relationship between cognitive workload and physiological responses does not straightforwardly correlate with cognitive demands as might be intuitively expected.

This suggests that physiological stress responses, as reflected in GSR and PR, are more closely tied to the demands of the teleoperation task itself rather than the cognitive complexity of working memory tasks. This aligns with prior findings, which suggest that such variables, along with breathing rate, are better indicative of accumulated fatigue over time^56^ rather than cognitive workload fluctuations within a task.

fNIRS was able to detect differences between n-back tasks in NT, however these differences were not observable during the high-workload primary teleoperation task in T. The analysis showed a clear pattern of brain activity in response to increasing mental workload from 0-back to 3-back tasks in NT, but not in T. Notably, moving from 0-back to 1-back tasks resulted in a decrease in HbO and an increase in HbR (the opposite to what is normally expected), indicating a significantly lower level of neural response to the 1-back task. The unexpected initial decrease in neural response from 0-back to 1-back might suggest that the 0-back condition engages participants differently, possibly due to its demand for continuous response (i.e., saying no to every stimulus) rather than a decisive response to stimuli. Anecdotally, we have found it hard in our past work to make sure participants do not mind-wander when doing near-zero effort repetitive tasks. This could lead to increased cognitive workload levels during 0-back, which then reduces when participants are engaged in a low workload 1-back task. As the tasks became more challenging (from 1-back to 3-back), the expected increases in HbO and decreases in HbR were observed, reflecting increased neural engagement and cognitive effort.

When comparing T and NT, only the 1-back task showed a significant increase in HbO and a decrease in HbR during teleoperation. This suggests that teleoperation introduces an additional layer of workload or requires a different cognitive strategy, as evidenced by the distinct neural activation patterns. Another explanation for this outcome could be that 1-back task is a balance between a task which is sufficiently engaging cognitively, but not overly demanding. This moderate level of difficulty, combined with teleoperation, might be enough to see a significant increase in workload. However, the same significant difference was not observed between T and NT for other levels of n-back task, implying again that fNIRS may not be sufficiently sensitive to distinguish already high-levels of cognitive workload.

These findings are relevant for ongoing research on using physiological data to adapt task demand in human-robot interaction. The lack of objective physiological measure differences between 1-back, 2-back, and 3-back conditions during teleoperation could be attributed to several factors. One possibility is the redistribution of cognitive resources, where individuals allocate cognitive capabilities in a way that task difficulty differences do not alter their measured cognitive workload. Another possibility is that fNIRS may not have the necessary sensitivity to detect subtle variations in cognitive workload during high workload tasks like teleoperation. Furthermore, teleoperation involves physical movements, which can introduce motion artefacts into fNIRS data, affecting brain signal quality and making it challenging to accurately measure brain activity related to teleoperation. Therefore, considering the complexity of the observed neural responses, the potential for motion artifacts inherent in teleoperation tasks, and the limitations in sensitivity to discrete variations in cognitive workload, our analysis might suggest that while fNIRS may provide useful insights in certain contexts, it may not be ideal for measuring nuanced workload variations in teleoperation.

### Implications for teleoperation and human-machine interface

Increased cognitive workload negatively impacted teleoperation effectiveness across several dimensions, including remote end-effector speed, power usage, and energy expenditure, while robot motion smoothness remained largely unaffected.

Significant differences in performance indicators, particularly between the least and most demanding cognitive conditions, underscore how elevated cognitive demands compromise teleoperation. Operators facing higher workload levels may prioritise precision over speed to reduce errors, a conservative strategy often observed in various domains under increased cognitive demands. Significant differences in power and energy consumption, with lower values in higher n-back conditions, suggest that operators adopt slower, more efficient maneuvers when under cognitive strain, further indicating a cautious approach. The consistency in robot motion smoothness across cognitive workload levels may reflect either resilience in basic motor skills required for teleoperation, or a limitation in the sensitivity of smoothness measures used in the study.

Our findings suggest that a combination of subjective reports and teleoperation performance indicators offers the most robust tools for measuring workload in teleoperation. These methods provide insights into both the operator’s subjective experience and the challenges faced during the task. These insights are essential for designing teleoperation systems and interfaces for human-machine interaction. They reinforce existing research emphasizing that teleoperation interface design critically influences workload, hence affect operator performance. Misalignment in interface design can increase workload, potentially compromising operational efficiency and increasing cognitive strain on operators^57^. Therefore, interface design should aim to minimize workload and stress by observing, measuring and responding to how users interact with teleoperation tasks and adapting to users’ needs and responses. In addition, strategies such as incorporating partial automation and prioritizing interface consistency, reliability, and transparency could enhance operator satisfaction and efficiency by reducing the cognitive demands of robot teleoperation and facilitating behavior prediction.

### Limitations of the study

This study presents findings that should be considered alongside certain limitations related to participant experience, setup complexity, and metric scope. The participant pool, primarily consisting of University of Nottingham students and staff with limited teleoperation experience, may not fully represent the real-world settings. Although participants received training and participated in practice sessions before the experimental trials, experienced teleoperators with substantial field experience may exhibit different operational patterns and physiological responses, having developed skills and strategies to manage cognitive and physical demands more effectively.

Additionally, the experimental setup was basic, involving only two Franka Emika robotic arms separated by a curtain. This simple arrangement does not reflect the complexity of real-world teleoperation control rooms, which often feature advanced information systems, sophisticated control interfaces, complex visual displays, and strict supervision protocols. These additional elements could significantly impact teleoperator performance and workload, aspects not captured in this study.

Finally, the study focused on a limited range of performance and physiological metrics. Examining a broader set of metrics could offer a more comprehensive understanding of teleoperation under different workload levels. Additional performance measures, such as path efficiency, along with further physiological metrics, such as gaze tracking, detailed cardiovascular responses, and muscle activity through electromyography (EMG), could provide deeper understanding around the impacts of workload on teleoperation.

## Conclusion

In teleoperation, where multitasking is expected under critical conditions with little freedom to switch between primary tasks and accompanying sub-tasks, it becomes crucial to investigate how workload varies with primary and secondary task demands. Most existing studies in teleoperation have focused extensively on measuring cognitive workload and physical workload relating to feedback limitations, such as latency in sensory feedback. This study is a first to explore the cognitive and physical aspects of teleoperation separately in a controlled factorial design study, aiming to identify how high workload levels from various sources can be detected. An extended goal of this work is to investigate how we can dynamically manage task demands, offering tailored support when users experience high workload.

Our results clearly show that both the primary task (teleoperation) and the secondary task (working memory) impacted performance and subjective perceptions of workload. Physiological measures related to galvanic skin response and cardiovascular activity showed significant differences in different levels of the primary task but remained unaffected by the secondary task demand. Finally, fNIRS successfully detected changes in cognitive workload for different levels of the n-back working memory task, but not for workload variations during teleoperation.

Among the approaches investigated during this study, task performance measurements were most robust to recognising variations in task demand during a high mental workload teleoperation task. Although it is possible that a larger study sample may reach levels of significance, future research could explore additional objective physiological measures like breathing patterns, pupil dilation, and facial thermography, to see if the changes in these are observable across different levels of workload during teleoperation.

## Data Availability

The datasets used and/or analysed during the current study are available from the corresponding author upon reasonable request.
